# Development of an Innovative Dual Construct for Targeted Drug Delivery in the Oral Cavity

**DOI:** 10.3390/pharmaceutics17020272

**Published:** 2025-02-18

**Authors:** Elena Mazzinelli, Ilaria Favuzzi, Marianna Messina, Giorgia Fratocchi, Federica Vincenzoni, Eleonora Santo Stefano, Francesco Cecconi, Carlo Lajolo, Alessia Basco, Raffaella Castagnola, Massimo Cordaro, Francesco Scilla, Valerio Papa, Alessandro Arcovito, Ilaria Cacciotti, Giuseppina Nocca

**Affiliations:** 1 Dipartimento di Scienze Biotecnologiche di Base, Cliniche Intensivologiche e Perioperatorie, Università Cattolica del Sacro Cuore, Largo Francesco Vito 1, 00168 Rome, Italy; elena.mazzinelli@unicatt.it (E.M.); ilaria.favuzzi@gmail.com (I.F.); giorgia.fratocchi@yahoo.com (G.F.); federica.vincenzoni@unicatt.it (F.V.); santostefano.1641637@studenti.uniroma1.it (E.S.S.); francesco.cecconi@unicatt.it (F.C.); 2 Dipartimento di Ingegneria, INSTM RU, Università degli Studi Niccolò Cusano, via Don Carlo Gnocchi 3, 00166 Rome, Italy; marianna.messina@unicusano.it (M.M.); valerio.papa@unicusano.it (V.P.); ilaria.cacciotti@unicusano.it (I.C.); 3 Fondazione Policlinico Universitario A. Gemelli IRCCS, Largo Agostino Gemelli 8, 00168 Rome, Italy; 4 Dipartimento di Testa-Collo ed Organi di Senso, Università Cattolica del Sacro Cuore, 00168 Rome, Italy; carlo.lajolo@unicatt.it (C.L.); alessiabasco19@gmail.com (A.B.); raffaella.castagnola@unicatt.it (R.C.); massimo.cordaro@unicatt.it (M.C.); francesco.scillaod@gmail.com (F.S.); 5 UOC di Clinica Odontoiatrica, Dipartimento di Neuroscienze, Organi di Senso e Torace, Fondazione Policlinico Universitario A. Gemelli IRCCS, 00168 Rome, Italy

**Keywords:** poly(lactic acid) fibers, poly(lactic-*co*-glycolic acid) nanoparticles, chitosan coating, dexamethasone, mucoadhesivity, drug delivery, oral cavity

## Abstract

**Background:** Oral lichen planus (OLP) is a chronic autoimmune disease of the oral mucosa, classified among potentially malignant oral disorders (OPMDs). It is characterized by keratinocyte apoptosis and persistent inflammation. Standard treatments involve topical corticosteroids administered via mouthwashes, gels, or ointments, but these require frequent application, have limited retention, and may cause side effects. To address these limitations, this study aimed to develop an innovative dexamethasone delivery system targeting the oral cavity, based on poly(lactic acid) (PLA) fibers coated with chitosan (CS) and poly(lactic-*co*-glycolic acid) (PLGA) nanoparticles. **Methods:** CS-coated PLA fibers were characterized for their mucoadhesive and cytocompatibility properties, while PLGA nanoparticles were analyzed for size, shape, encapsulation efficiency, cellular uptake, drug release efficiency, and cytocompatibility. **Results:** Both polymers demonstrated cytocompatibility, and chitosan-coated PLA fibers exhibited mucoadhesive properties. PLGA nanoparticles were effectively internalized by the cells and successfully released the drug into the cytoplasm. The combination of CS-coated PLA fibers and PLGA nanoparticles provided dual benefits: mucoadhesion and efficient cellular uptake, even under conditions simulating salivation. **Conclusions:** These findings highlight the potential of the proposed system to improve mucoadhesive drug delivery. Further optimization is needed to enhance patient compliance and therapeutic efficacy.

## 1. Introduction

Oral potentially malignant disorders (OPMDs) are pathological conditions which may evolve into oral squamous cell carcinoma (OSCC) [1]. Among OPMDs, oral lichen planus (OLP) shows a malignant transformation rate equal to 1.4–2.28% [2,3], even if this percentage is clearly underestimated due to restrictive diagnostic criteria and inadequate follow-up of patients [2]. OLP is a chronic immunologically mediated mucocutaneous disease [4,5], which may affect only the oral cavity. The main risk factors linked with malignant transformation include the atrophic–erosive phenotype, tongue involvement, female gender, and advanced age, emphasizing the importance of regular oral cancer clinical–histological screenings [6]. It is evident that, despite the well-established risk of cancer development, there remains a significant gap in knowledge regarding the behavior, prognosis, and clinical characteristics of OSCC developing from OLP [7].

**Figure 1:**
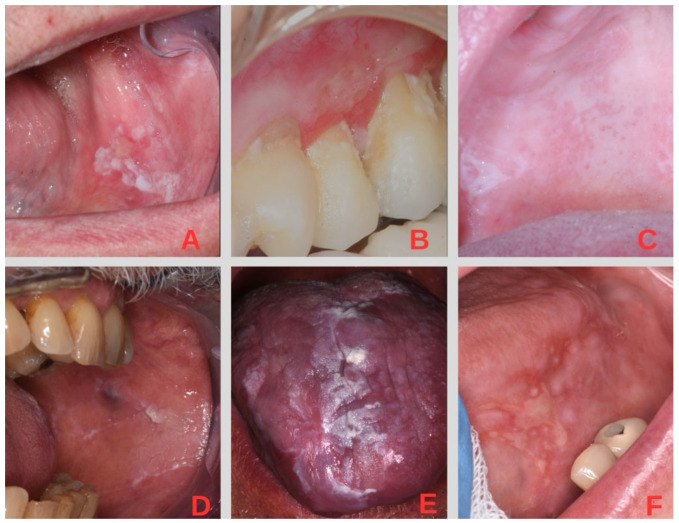
Examples of the different clinical manifestations of OLP. (**A**) Plaque-like lichen. (**B**) Atrophic lichen. (**C**) Reticular lichen. (**D**) Melanin-related hyperpigmentation from lichen. (**E**) Verrucous plaque-like lichen. (**F**) Ulcerative lichen.

The global pooled prevalence of OLP is about 1%, and it is more common in middle-aged people, with a prevalence that progressively increases [5] and shows a preference for the female gender [8,9]. Characteristic clinical features of OLP include symmetric, bilateral, or multiple distributions of looping white lines or striae that are usually located in the buccal, labial, and gingival mucosae; margins; and the back of the tongue. Two different groups of clinical patterns are recognized: erosive–atrophic forms (including atrophic–erosive and bullous forms) and non-erosive–atrophic forms (including reticular, papular, and plaque-like forms) [10] (Figure 1).

The precise etiology of OLP is unknown but an important role seems to be played by cell-mediated immunity. According to this hypothesis, the first event of OLP could be keratinocyte antigen exposure causing a cytotoxic CD8^+^ cell response [11]. Both CD4^+^ helper cells and CD8^+^ cytotoxic T cells are activated in OLP. Moreover, alterations in T lymphocyte autophagy, leading to the dysregulation of apoptosis in oral keratinocytes, have been reported in OLP patients [12].

Several OLP forms are asymptomatic or paucisymptomatic, requiring just periodic follow-up. On the other hand, atrophic–erosive OLP can often cause symptoms, ranging from a mild burning sensation to severe pain, significantly compromising the patient’s quality of life [13]. This form often requires a pharmacological treatment, aimed at reducing local immune–inflammatory reaction and thus the possible malignant transformation risk [3].

The gold standard for OLP treatment is represented by the use of pharmaceutical formulations, based on topical corticosteroids, as well as calcineurin inhibitors (e.g., Tacrolimus and Cyclosporine [14]). The most-used corticosteroids are dexamethasone (DEX) and clobetasol propionate [15,16], which exert an anti-inflammatory effect, reducing the number of immune cells and the production of cytokines at the inflammation site [15]. However, the long-term use of topical corticosteroids may cause adverse effects. The systemic side effects include adrenal suppression, gastrointestinal upset, hypertension, and hyperglycemia, and the local ones embrace thinning of the oral mucosa, developing secondary candidiasis, and refractoriness [17,18,19]. As of today, there are a few formulations specifically designed for the oral cavity, while most are derived from dermatological applications. This is because, despite the widespread use of the oral mucosa as a site for drug administration, it presents several challenges due to its specific anatomical and functional characteristics, such as constant movement (e.g., speaking and chewing), saliva secretion, and the swallowing reflex, which reduce the retention time of drug delivery systems. Additionally, the development of compounds tailored for oral mucosal delivery is limited by the lack of standardized in vivo and in vitro models capable of accurately predicting and quantifying drug effects and potential issues. This gap in predictive systems hinders the development and market availability of new formulations [20].

To overcome these limitations, it is necessary to improve the adhesiveness of drug delivery systems to the oral mucosa, ensuring prolonged contact and increasing retention time within the oral cavity. These improvements would enhance drug bioavailability and optimize therapeutic outcomes, addressing current challenges in this field [21,22,23].

For this reason, topical corticosteroids such as betamethasone valerate, clobetasol propionate, fluocinolone acetonide, fluocinonide, and triamcinolone acetonide have been incorporated into formulations of various adhesive pastes, which are characterized by good bioadhesive and delivery properties [24]. However, thanks to the development of nanotechnologies, it is now possible to further improve the mucoadhesive properties and the duration of interaction with the mucosa by using selected drug delivery systems (DDSs), such as nanoparticles (NPs) and fibers (FBs) coated with mucoadhesive molecules (e.g., chitosan) [25,26,27]. For instance, innovative materials such as metal–organic frameworks (MOFs) and graphene oxide-based nanoparticles have been explored for their potential in enhancing drug delivery, particularly in the context of oral cavity applications [28]. Recent advances in nanomaterials, including polythiophene-based systems, offer exciting possibilities for targeted therapy and improved biocompatibility in oral mucosal applications [29]. Nanofiber-based drug delivery systems, especially those incorporating core–shell structures, have shown promise in controlled drug release, which could further enhance the effectiveness of treatments in the oral cavity [30]. Poly(lactic-*co*-glycolic acid) (PLGA) and poly(lactic acid) (PLA) are two of the most widely used and promising biopolymers for the development of drug delivery systems [31,32]. Both PLGA and PLA are biodegradable, and their degradation products, lactic acid and glycolic acid, are metabolized in the body through pathways linked to the Krebs cycle [33]. Lactic acid is primarily converted back to pyruvate by the enzyme lactate dehydrogenase in muscles and other tissues. Pyruvate then enters the Krebs cycle to produce energy. Additionally, lactic acid can be transported to the liver, where it is converted into glucose via the Cori cycle, which is then reused by the body for energy. Glycolic acid, on the other hand, is oxidized to glycolate, which can be further converted to oxalate. Oxalate is excreted by the kidneys in the urine, although some glycolate can also be metabolized into compounds that enter the Krebs cycle for energy production. Consequently, the systemic toxicity of PLGA and PLA is negligible.

In this study, we aimed to address the challenges of drug delivery in the oral cavity by designing and developing an innovative drug delivery system tailored for oral lichen planus (OLP). The proposed system combines PLA fibers coated with chitosan and PLGA nanoparticles to enhance mucoadhesion and ensure prolonged drug retention in the oral cavity, respectively. To validate this system, we utilized a bioreactor simulating salivary flow and OLP biopsy-derived cells cultured on a PLA scaffold, recreating key aspects of the 3D structure of the oral mucosa. This approach seeks to bridge the gap between in vitro and in vivo models, providing a novel platform to improve therapeutic outcomes for OLP patients.

## 2. Materials and Methods

### 2.1. Materials

Unless otherwise indicated, all materials used in this work were obtained from Sigma Aldrich (Milan, Italy) and used as received. Polylactic acid (PLA) (3051D; density of 1.25 g/cm^3^, average molecular weight numerical (Mn) of approx. 1.42 × 10^4^ g/mol) was purchased from Nature Work (Minneapolis, MN, USA), and chloroform (CHCl_3_, 99%), N-Dimethylformamide (DMF; (CH_3_)_2_NOCH, 99.8%), and acetic acid (C_2_H_4_O_2_) were obtained from VWR International. Acetonitrile (CH_3_CN, HPLC-grade; VWR chemical, Milan, Italy) and ultrapure water (obtained by a P.Nix Power I System apparatus; Human Corporation, Seoul, Republic of Korea) were used for High-Performance Liquid Chromatography (HPLC) analysis.

### 2.2. Methods

#### 2.2.1. Human Fibroblast Isolation and Cell Cultures

Human fibroblasts (HFs) were obtained from biopsies of OLP patients (authorization granted by the UCSC Ethics Committee—prot. ID: 4581; 14 December 2021), as reported in [34]. HFs were grown in Dulbecco’s modified Eagle’s medium (DMEM) supplemented with 10% fetal bovine serum (FBS), penicillin (100 units/mL), streptomycin (10.0 mg/mL), amphotericin B (2.5 mg /mL), and L-glutamine (2 mmol/L) at 37 °C, under 5% CO_2_ conditions. All experiments were performed using cells from the third to the fifth passage because beyond these passages cells may undergo changes such as differentiation, senescence, or loss of specific markers, which can affect the reliability of experimental results.

3T3-Swiss mouse fibroblasts, a cell line recommended by ISO standards for biomaterial studies [35] (kindly provided by Prof. Giovan Battista Pani, UCSC) were cultured at 37 °C in a humidified atmosphere with 5% CO_2_, in DMEM supplemented with 10% FBS, penicillin (100 units/mL), streptomycin (10.0 mg/mL), and L-glutamine (2 mmol/L).

#### 2.2.2. Evaluation of Cytotoxicity Induced by DEX Without a Carrier (DEX-f)

3-(4,5-Dimethylthiazol-2-yl)-2,5-Diphenyltetrazolium Bromide (MTT) assays were performed using free DEX (unencapsulated, f-DEX) to establish the non-cytotoxic concentration for subsequent delivery.

For this purpose, 3T3-Swiss fibroblasts were treated with decreasing concentrations of f-DEX. A stock solution of the drug was freshly prepared before use, with DEX dissolved in dimethilsuphoxy acid (DMSO) at various concentrations ranging from 0.003 to 0.1 mg/mL.

The final concentrations of DEX were as follows: 100 µg/mL, 50 µg/mL, 25 µg/mL, 6 µg/mL, and 3 µg/mL.

The final concentration of DMSO, used as the vehicle, remained consistent in all samples during the experiments (0.1% *v*/*v*).

To conduct the assay, 3T3-Swiss fibroblasts were plated in a 96-well tissue culture dish at a density of 1 × 10^4^ cells/well in DMEM. After 24 h of incubation, the medium was removed and the cell monolayer was incubated with DEX at the final concentrations mentioned above. After another 24 h of incubation, cell viability was assessed using the MTT assay, as described in [36]. Briefly, 20 µL of MTT solution (5 mg/mL in PBS) was added to each well containing 200 µL of medium, resulting in a final 1:10 dilution of the MTT solution. After 4 h of incubation at 37 °C, the produced intracellular formazan crystals were solubilized with an isopropanol solution containing HCl (4 × 10^−2^ M, 0.50 mL). The absorbance (Abs) of the solutions in each well was measured using an automatic microplate photometer (ELx800; BioTek, Bad Friedrichshall, Germany) at a wavelength of 562 nm. Each experiment was performed in sextuplicate and repeated three times on different days independently; the cytotoxicity was calculated using the following equation [36]:

% Cell Viability = [(Abs Sample)/(Abs Control)] × 100.(1)

Specimens were classified as slightly, moderately, or severely cytotoxic when cell viability, relative to controls, was >70%, between 70% and 40%, or <40%, respectively [37].

#### 2.2.3. HPLC Analysis

To evaluate the concentration of DEX inside the cells, HPLC analysis was performed using a system composed of a Thermo Finnigan MS pump, a Photodiode Array (PDA) detector, and an autosampler. A C-18 (3 µm) Supelco reversed-phase column measuring 150 mm × 4.7 mm was employed for chromatographic separation.

The mobile phase was composed of 50% CH_3_CN and 50% distilled water (dH_2_O). All measurements for DEX were conducted at a wavelength (λ) of 254 nm [38]. The analyses were carried out using an isocratic mode with a constant flow rate of 0.3 mL/min and a standard injected volume of 100 µL. Each analysis was performed in quintuplicate to ensure the robustness of the results. Calibration curves were constructed by diluting the active ingredients in dH_2_O at various concentrations ranging from 0.625 to 5 µg/mL.

Additionally, the absorbance for each concentration (expressed as Area Under the Curve—AUC) was determined. To define a standard and therefore a calibration curve, quintuplicate measurements of each concentration were performed, and the means and standard deviations of the recorded absorbances were calculated. Using the obtained curves, the limit of quantification (LOQ) and the limit of detection (LOD) were determined:

LOD = (3.3 × SD/slope)

LOQ = (10 × SD/slope)

In addition to the calibration process, recovery studies were conducted to evaluate the efficiency of drug recovery from cell lysates. For this purpose, two different concentrations of DEX, i.e., 1.85 μg/mL and 1.72 μg/mL, were added to the cell lysates, with two replicates prepared for each concentration. To this end, 3T3-Swiss fibroblasts were seeded in 75 cm^2^ flasks. When the cells reached sub-confluence, the DMEM was removed from each flask and the cell monolayers were washed with PBS and lysed by freezing at −80° [39]. Cellular lysates were resuspended in 4 mL of water, and the aforementioned amounts of DEX were added. All samples were centrifuged at 15,000 rpm for 10 min at 4 °C, and the supernatants were collected, filtered using a 0.22 µm filter, and loaded into the HPLC column. The absolute recovery was assessed by calculating the ratio between the experimentally observed concentration and the theoretical concentration.

As a control, the analyses were also performed without DEX administration.

#### 2.2.4. Preparation and Characterization of Polylactic Acid and Chitosan–Polylactic Acid-Based Fibers (PLA-FBs and CS-PLA-FBs)

PLA fibers (PLA-FBs) were prepared using the electrospinning technique [40]. PLA pellets (15% *w*/*v*) were dissolved in a mixture of CHCl_3_ and DMF (67:33 *v*/*v*) under constant magnetic stirring (150 rpm) at approximately 40–50 °C for 2 h to promote the complete dissolution of the polymer. Afterwards, the solution was cooled to room temperature and kept under agitation overnight before electrospinning.

Subsequently, the prepared solution was poured into a glass syringe equipped with an 18 G needle, fixed on an infusion pump (KDScientific, Holliston, MA, USA), and various parameters were set up: voltage: 12 kV (Spellman model SLM50P300), needle-collector distance: 15 cm, flow rate: 0.5 mL/h. After deposition on the collector, the membranes (PLA-FBs) were detached and allowed to dry at room temperature for 24 h.

To provide mucoadhesive properties, the obtained fibrous mats were dipped into an aqueous acetic acid (C_2_H_4_O_2_) (1% *v*/*v*) solution of low-molecular weight chitosan powder (50–190 kDa, with a 75% deacetylation degree) (2% *w*/*v*).

The morphology of the PLA-FBs was observed using Scanning Electron Microscopy (SEM; Zeiss SUPRA 25; Zeiss, Jena, Germany). The PLA-FBs were placed on aluminum supports (stubs), to which conductive carbon adhesive disks were applied. The samples were then coated with a 50 nm thick gold film using a high-resolution sputter coater (Agar Scientific B7234; Agar Scientific, Rotherham, UK), setting a density of 19.30 g/cm^3^ and 40 mA/s. For the measurement of diameters using ImageJ 2.9.0/1,53 t, 100 fibers were randomly selected by analyzing SEM images captured at magnifications specifically chosen to minimize human error. The magnifications used ranged between 10,000× and 20,000× to ensure a balance between resolution and precision in the measurements. Following the importation of the SEM images into ImageJ, the scale bar provided by the SEM was utilized to set the scale. The ‘Sraight’ tool in ImageJ was then employed to measure the diameters of the fibers by manually tracing segments perpendicular to the longitudinal axis of the fibers. To ensure a representative sample, SEM images were captured from different positions of the sample, and the fibers were randomly selected from these images. This approach supports the confidence that the selection of 100 fibers provides a statistically meaningful basis for characterizing the sample. Histograms and the relative cumulative curves were reconstructed using Origin software 2019b 32bit.

Moreover, infrared spectroscopy measurements were carried out on both uncoated and CS-coated PLA fibers at the spectral resolution of 4 cm^−1^, using an FT-IR instrument equipped with an attenuated total reflectance (ATR) cell (Nicolet iS5), in the following conditions: region: 600–4000 cm^−1^, scan number: 16, spectral resolution: 4 cm^−1^.

#### 2.2.5. Preparation and Characterization of DEX-Loaded Poly(lactic-*co*-glycolic) Nanoparticles (DEX-PLGA-NPs)

The PLGA-NPs were prepared with a single emulsion followed by the solvent evaporation method [30]. Briefly, for organic-phase preparation, 50 mg of PLGA (L-lactide/glycolide: 75:25, molecular mass: 66–107 kDa) and 5 mg of DEX were dissolved in 3 mL of organic solvent, acetone, and dichloromethane 1:1 (*v*/*v*). The organic phase was emulsified to an aqueous phase (PVA 1.0% *w*/*v* in 7 mL of dH_2_O) through sonication with a vortex (Mini Vortexer; VWR, West Chester, PA, USA) at 3200 rpm for 1 min, adding the organic solution drop by drop to the aqueous solution. The emulsion was then sonicated in ice for 1 min—ampl.: 45%, pulse: 10 s, pause: 5 s—using a Vibra cell sonicator (Bioblock Scientific, Illkirch, France). To allow organic-phase evaporation, the emulsion was stirred (700 rpm for 3 h) in the dark at room temperature. Once synthesized, the DEX-PLGA-NPs were washed twice in dH_2_O using an ultracentrifuge (Avanti J-25; Beckman, Cassina de´Pecchi (Milano, Italy) (15,700 rpm for 50 min at 10 °C). The obtained pellet was resuspended in 10 mL of dH_2_O and analyzed by Dynamic Light Scattering (DLS) (DLS; Zetasizer Nanoseries ZS90, Malvern Instrument, Malvern, UK). The resulting suspension was frozen at −20 °C and finally freeze-dried using the VirTis benchtop freeze dryer 6 K (VirTis, Gardiner, NY, USA). The PLGA-NPs’ morphology was examined by SEM. A droplet of the aqueous phase containing PLGA-NPs was placed on an aluminum stub covered with a conductive carbon disk, which was then dried and metallized with a 40 nm thick gold film using a sputter coater (High Resolution Sputter Coater AGB7234 Agar Scientific, Rotherham, UK) (19.30 g/cm^3^ and 40 mA/s) before analysis by SEM. Observations were performed at 100–1000×.

The production efficiency (PE) was calculated according to Equation (2) [31]:

PE (%) = [(mg of dried PLGA-NPs)/(mg of initial PLGA)] × 100.(2)

The load evaluation of DEX in the PLGA-NPs was measured by HPLC analysis. A quantity of 2 mg of lyophilized PLGA-NPs was dissolved in 2 mL of CH_3_CN and dH_2_O 95:5 (*v*/*v*) under magnetic stirring (700 rpm). Then, the sample was centrifuged at 10,000 rpm for 10 min, and 1 mL of the supernatant was analyzed by HPLC. The DEX loading concentration was evaluated using a calibration curve obtained in a concentration range of 1.25 µg/mL to 10 µg/mL.

The entrapment efficiency (EE) percent was calculated according to Equation (3) [9]:

EE (%) = [(mg of drug incorporated)/(mg of drug initially taken)] × 100.(3)

All experiments were performed in triplicate.

To confirm the absence of interaction between DEX and PLGA, in vitro release studies of DEX from PLGA-NPs were performed in EtOH. A fixed mass of lyophilized PLGA-NPs (1.3 mg, equivalent to 140 μg of DEX) was suspended in 2 mL of EtOH at 37 °C and stirred at 300 rpm. At predetermined time intervals (30 min, 4 h, 6 h, and 24 h), 1 mL of solution was withdrawn and replaced with 1 mL of fresh solvent to maintain a constant volume. The drug release was monitored by measuring Abs at 240 nm with a spectrophotometer (BECKMAN Coulter DU800; Brea, CA, USA) and compared with a calibration curve (3–100 μg/mL).

#### 2.2.6. Cellular Uptake of 6-Coumarin-Loaded PLGA-NPs

HFs were seeded in 24-well plates at a density of 2 × 10^4^ cells per well and incubated for 24 h to facilitate cell attachment. The cells were then treated for 60 min with 6-coumarin-loaded NPs (100 μg/mL), synthesized using the same method as the DEX-loaded NPs described in Section 2.2.5. Subsequently, imaging was performed using a fluorescence microscope (Zeiss Axio Observer; Zeiss, Jena, Germany) equipped with an Axio Cam 305 color camera.

#### 2.2.7. Effect of CS-PLA-FBs and DEX-PLGA-NPs on Cell Viability

The cytotoxicity of CS-PLA-FBs and DEX-PLGA-NPs was evaluated using the MTT assay. This test was conducted on both 3T3-Swiss fibroblasts and HFs. Cells were initially seeded into a multi-well plate, and, after 24 h of incubation, CS-PLA-FBs and PLGA-NPs were added according to specific conditions. The cells were treated with empty CS-PLA-FBs and with PLGA NPs loaded with DEX at concentrations of 50 μg/mL, 100 μg/mL, 150 μg/mL, and 200 μg/mL. The control group consisted of untreated cells.

The multi-well plate was then incubated for an additional 24 h, after which the MTT assay was performed as described above [36].

#### 2.2.8. In Vitro Release Studies of DEX from PLGA-NPs and Determination of Intracellular Concentrations

PLGA-NPs were deposited onto cells (initially on 3T3-Swiss fibroblasts to assess the protocol and then on HFs). DEX-loaded PLGA-NPs were added to the culture medium (10 mL) in a quantity sufficient to provide a DEX concentration of 70 µg/mL. The release of DEX was analyzed after 4 h of incubation with cells at 37 °C though HPLC analysis. As controls, empty PLGA-NPs and a DEX-free (DEX-f) solution at a concentration of 70 µg/mL were used.

After incubation, the cells were washed with PBS and lysed by freezing at −80 °C. Cellular lysates were resuspended in 4 mL of water and centrifuged at 15,000 rpm for 10 min at 4 °C. The supernatant was filtered through a 0.22 µm filter and analyzed by HPLC [39].

#### 2.2.9. Mucin–CS-PLA-FB Interaction

The mucoadhesiveness of the CS-PLA-FBs was evaluated using the modified Periodic Acid–Schiff (PAS) assay by immersing 2 mg of CS-PLA-FBs in 5 mL of PBS (pH 6.5).

Two different concentrations (0.4 and 2.0 mg/mL) of mucin, derived from porcine stomach type II, were prepared by resuspending it in PBS. The mucin solutions were then added to the CS-PLA-FBs in PBS. The mixtures were incubated at 37 °C for 90 min to allow interaction between the CS-PLA-FBs and the mucin.

Control experiments were conducted by incubating CS-PLA-FBs in PBS without mucin and by incubating PLA-FBs with the mucin solutions, under the same conditions. After the incubation period, the fibers were carefully removed from the mucin solutions and washed thoroughly in PBS to remove any non-adhered mucin.

The washed fibers were immersed in 5 mL of a 0.3% PAS solution containing 350 µL of glacial acetic acid. This mixture was incubated at 37 °C for 2 h. The periodic acid treatment was performed to oxidize the mucin molecules, rendering them reactive to the Schiff’s reagent in the following staining step.

Following the PAS treatment, Schiff’s reagent was added to the specimens, and then they were incubated at room temperature for 30 min. The Schiff’s reagent reacted with the oxidized mucin, producing a red–violet coloration indicative of the presence of mucin on the CS-PLA-FB surfaces.

At the end of the procedure, the coloration of the fibers was observed to compare the extent of mucoadhesion capacity across the different fiber types and mucin concentrations. It was expected that the CS-PLA-FBs, due to the presence of chitosan, would exhibit stronger mucoadhesion compared to the PLA-FBs, which did not contain chitosan.

Additionally, some samples of CS-PLA-FBs submitted to the periodic PAS assay were observed via SEM to microscopically evaluate the mucoadhesion effect.

#### 2.2.10. PLGA-NP Integration into PLA-FBs and Cellular Uptake

A PLA-FB membrane (Ø: 5 cm) was decorated with 6-coumarin-loaded-PLGA-NPs, applying the following procedure. A suspension of 20 mL (1 mg/mL) of the coumarin-loaded PLGA-NPs in dH_2_O was pumped though the PLA-FBs using a vacuum filtration device (Merck Millipore, Darmstadt, Germany). The PLA-FBs decorated with coumarin-loaded NPs were then exposed to 3T3-Swiss fibroblasts for 1 h. After this period, the fluorescence inside the cells was determined using fluorescence microscopy. A portion of the NP-decorated fibers was observed via SEM and submitted to infrared spectroscopy (FTIR/ATR) analysis in the following conditions: region: 600–4000 cm^−1^, scan number: 16, spectral resolution: 4 cm^−1^.

#### 2.2.11. Test in Bioreactor

**Figure 2:**
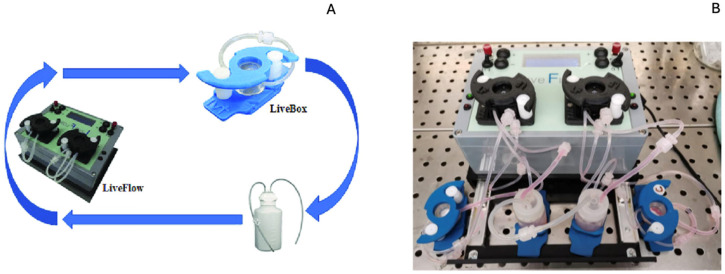
(**A**) Schematic representation of how the bioreactor works. (**B**) Live demonstration of the live boxes connected to the LiveFlow system.

To partially address the challenges of DDSs within the oral cavity, the main tests were repeated in a bioreactor that simulated salivary flow, with HFs grown in 3D inside the bioreactor to mimic the three-dimensionality of the mucosa without the cellular complexity. The bioreactor consisted of a Live Flow peristaltic pump (IV Tech, LF, Pisa, Italy), which uses peristaltic motion to transport fluids with two independent circuits and a flow rate between 100 and 450 µL/min, and two cell culture chambers, Live Box 2 (IV Tech, LB2), which were simultaneously perfused (Figure 2).

#### 2.2.12. Production of 3D PLA Scaffolds

**Figure 3:**
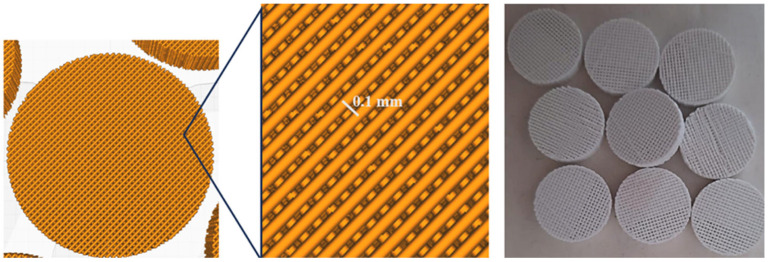
PLA scaffold design.

The experimental scaffolds were fabricated by Fused Deposition Modeling (FDM) 3D printing technology, using a PLA filament (Filoalfa, white, Ozzero, Italy). Specifically, the Creality Ender 3 Pro printer with Cartesian coordinates and a Bowden extruder (with the feed motor detached from the moving body of the head) was used. Cylindrical samples with a diameter of 1.4 cm and a height of 3.5 mm (Figure 3) were designed and manufactured on Autodesk Fusion CAD software 360, exported to the Ultimaker slicing software Cura version 5.2, and finally printed in the following conditions: nozzle of 0.2 mm, printing temperature of 200 °C, feed flow of 80%.

#### 2.2.13. Cell Proliferation on PLA Scaffolds

The optimal conditions for growing cells on PLA scaffolds were assessed first with 3T3 Swiss Fibroblasts and then with HFs. 3T3-Swiss fibroblasts were seeded onto the scaffolds at concentrations of 10.000, 25.000, and 50.000 cells per well using a 24-well tissue culture plate. Prior to cell seeding, the scaffolds were sterilized by immersion in EtOH and exposed to UV irradiation for 30 min at 254 nm to prevent microbial contamination [36].

MTT assays were conducted on the scaffolds after 7, 14, and 21 days, respectively. Specifically, the scaffolds were incubated with 1 mL of medium, followed by the addition of 100 μL of MTT solution. After 4 h of incubation at 37 °C, the solution was removed and 1 mL of isopropanol with HCl 0.04 M was added for 30 min. Subsequently, 200 μL of the acidic isopropanol solution from each well was transferred to a separate plate and analyzed as previously described. Each experiment was performed in triplicate and repeated three times (n = 3). To verify the absence of interference between the MTT assay and the scaffolds, the same procedure was conducted in the absence of cells.

Based on the results obtained with the 3T3-Swiss Fibroblasts, the aforementioned protocol was used to monitor HF proliferation: 25,000 fibroblasts were seeded onto each PLA scaffold, and the assay was conducted at 7, 14, and 21 days. Three technical replicates of each experiment were performed.

#### 2.2.14. DEX Releasing Test and Quantification of Intracellular Concentration

Based on the cell proliferation results, the release test proceeded as follows: 25,000 HFs were seeded onto a 3D PLA scaffold in a multi-well plate and incubated for 96 h to promote cell growth and adhesion. After this incubation period, the scaffold was transferred to the LB2 chamber in the bioreactor, where a flow rate of 400 μL/min was maintained. The experiment lasted 14 days, with the first 4 days under static conditions and the remaining 10 days under dynamic flow conditions to mimic a salivary flow environment. At the end of the 14-day period, PLGA nanoparticles were added to the scaffold in the LB2 chamber to achieve a DEX concentration of 70 µg/mL. After 4 h of incubation, the scaffold with cells was removed from the LB2, frozen at −80 °C, then resuspended in 2 mL of water and freeze-dried for analysis via HPLC.

### 2.3. Statistical Analysis

All results are reported as the means ± standard deviations (SDs), based on at least three separate experiments conducted in triplicates. The means were analyzed using analysis of variance (ANOVA), and if significant differences were found, a multiple comparison of means was performed using the Student–Newman–Keuls test. The significance level was set at 0.05.

## 3. Results

### 3.1. Evaluation of Cytotoxicity Induced by DEX-f

**Figure 4:**
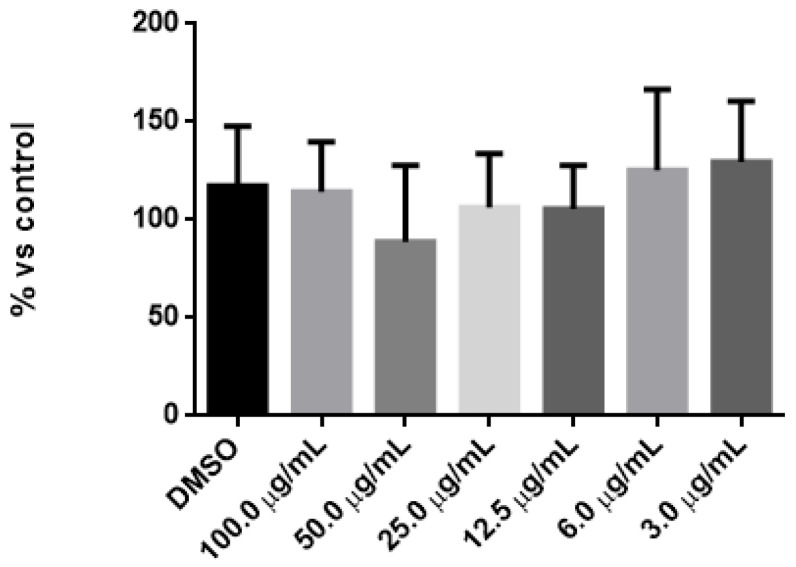
Cell viability after the treatment with the different concentrations of DEX-f in 3T3 Swiss Fibroblasts.

First, we evaluated the potential toxicity of our system. Figure 4 shows the results of the MTT analysis obtained by treating the cells with decreasing concentrations of free DEX (DEX-f). No toxicity was observed at the different concentrations, and no statistical differences were found between the DEX concentrations and DMSO (Table 1). We then proceeded with a DEX concentration of 70 µg/mL.

### 3.2. HPLC Analysis and Recovery Efficiency

**Figure 5:**
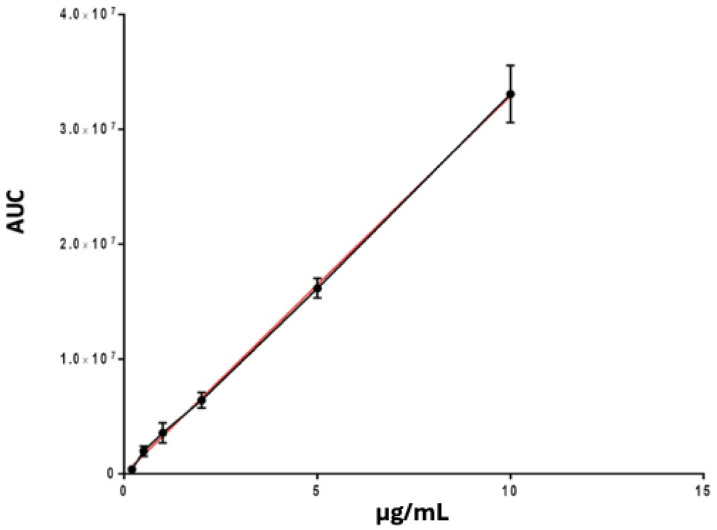
Calibration curve of DEX analyzed by HPLC. The red line is the linear regression curve used to calculate r^2^.

Since the chromatographic methods used were developed by modifying existing methods in the literature [38], it was necessary to first determine the LOQ and LOD values of the modified method before conducting the experimental tests. Additionally, prior to evaluating the drug recovery efficiency, the calibration curve was generated (Figure 5), and the regression equation and r^2^ value were determined. The obtained results are reported in Table 2.

According to this information, the drug recovery efficiency from the cell lysate was analyzed, amounting to 1.74 ± 0.08 µg/mL and corresponding to a 100% recovery efficiency (Table 3).

### 3.3. PLA-FB, CS-PLA-FB, and PLGA-NP Characterization

**Figure 6:**
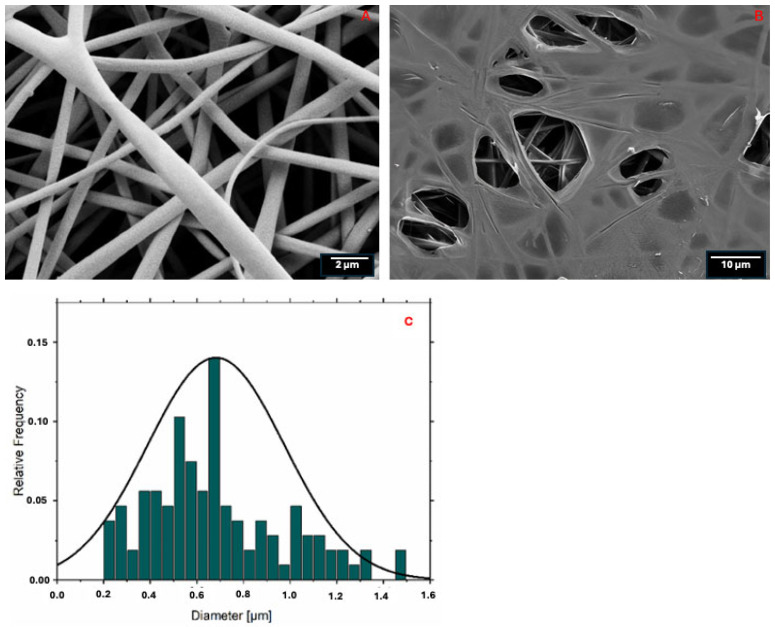
SEM micrographs of PLA-FBs. (**A**) Uncoated PLA-FBs. Scale bar: 2 µm. (**B**) PLA-FBs coated with chitosan. Scale bar: 10 µm. (**C**) Cumulative curves of the of diameter distributions of PLA-FBs.

**Figure 7:**
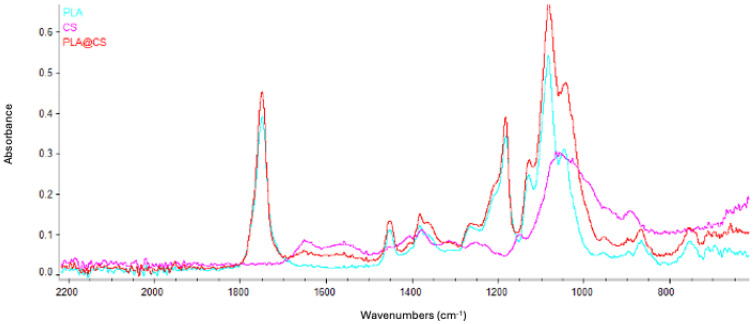
FTIR spectra of uncoated PLA fibers, CS-coated PLA fibers, and PLGA NPs.

Next, we analyzed the ultrastructure of the PLA-FBs. The morphology of PLA-FBs, uncoated and coated with CS, was observed using SEM (Figure 6). No surface defects were revealed, and, in the case of fibers coated with CS, it was possible to observe a surface layer with small interruptions, ascribable to the chitosan deposition (Figure 6B). A histogram (Figure 6C) demonstrates that the PLA-FBs typically have an average diameter measure ranging between 0.2 and 1.2 μm. The successful CS deposition on PLA fibers was further demonstrated by FTIR/ATR measurements (Figure 7). As evident from the comparison between the FTIR spectra of uncoated and CS-coated PLA fibers, in the case of coated ones, the co-presence of the PLA and chitosan typical vibrational modes was revealed, suggesting a physical adsorption. Indeed, no new peaks were detected due to the weak molecular interaction between chitosan and PLA chains [41].

As it concerned PLGA-NPs loaded with DEX, the dimensional characterization was performed using the DLS technique. This size was in line with previously reported values for PLGA-based nanoparticles used in drug delivery. For example, Ismail, R. et al. [42] reported nanoparticle sizes ranging from 160.1 ± 5.6 nm to 235 ± 5.3 nm, while Zhang, M. et al. [43] described sizes of approximately 102.6 ± 1.5 nm and 140.7 ± 0.6 nm.

**Figure 8:**
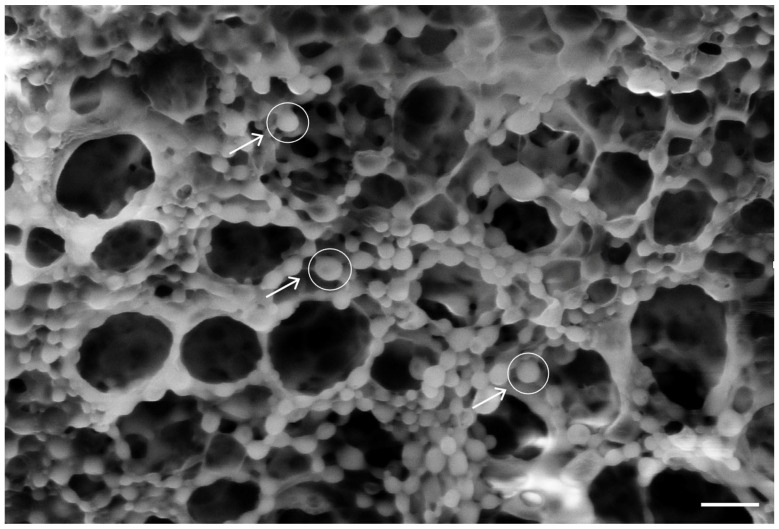
SEM micrograph of PLGA-NPs. The white line in the bottom right corner is the scale bar and corresponds to 1 µm. The white circles highlight the individual nanoparticles.

The synthesized nanoparticles had a Z-average value of 187 nm ± 22 nm. The predominant dimensional value was found to be 194 nm. The polydispersity index (PDI) of 0.09 and the narrow dimensions of the measured distributions also suggest that the sample contained a homogeneous particle suspension of spheroidal NPs with similar sizes. The spherical shape of the PLGA-NPs was further investigated using SEM (Figure 8).

After freeze-drying, the quantity of NPs obtained was equal to 25.7 mg ± 2.1 mg. The production efficiency of PLGA-NPs (evaluated using Equation (2)) was 51.3% ± 4.2%.

The loading quantity of the drug in 1 mg of lyophilized NPs was equal to 110 µg ± 6.2 µg, and the total amount of encapsulated drug was found to be equal to 2.9 mg ± 0.2 mg. Thus, the encapsulation efficiency was 57.0% ± 4.2% (according to Equation (2)).

Drug release tests from PLGA-NPs in EtOH highlighted that 100% of the drug is released after 1 h.

### 3.4. NP Cell Uptake: Internalization of 6-Coumarin

**Figure 9:**
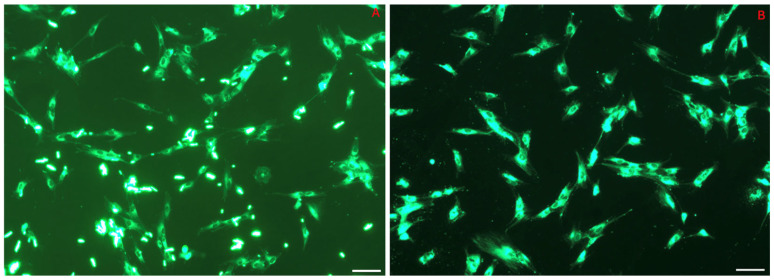
(**A**) Coumarin administered to HFs in a non-vehiculated form. (**B**) Coumarin administered via PLGA-NPs. The internalization of NPs in HFs was observed using a fluorescence microscope. Zoom: 20×. Scale bar: 50 µm.

The next step was to evaluate the cell uptake capacity of the system. Figure 9 illustrates the ability of PLGA-NPs to penetrate inside the HFs after 60 min of incubation. The image demonstrates the extent to which the PLGA nanoparticles—loaded with 6-coumarin—successfully entered and distributed within the HFs during the incubation period. The comparison between picture A (where the fluorescent probe was not delivered via a carrier) and picture B (where it was administered through PLGA-NPs) clearly shows that, in the latter case, the intracellular fluorescence was due to the entry of the NPs, as the medium was completely free of fluorescent signals, unlike what was observed in the first condition.

### 3.5. Effect of CS-PLA-FBs and DEX-PLGA-NPs on Cell Viability

**Figure 10:**
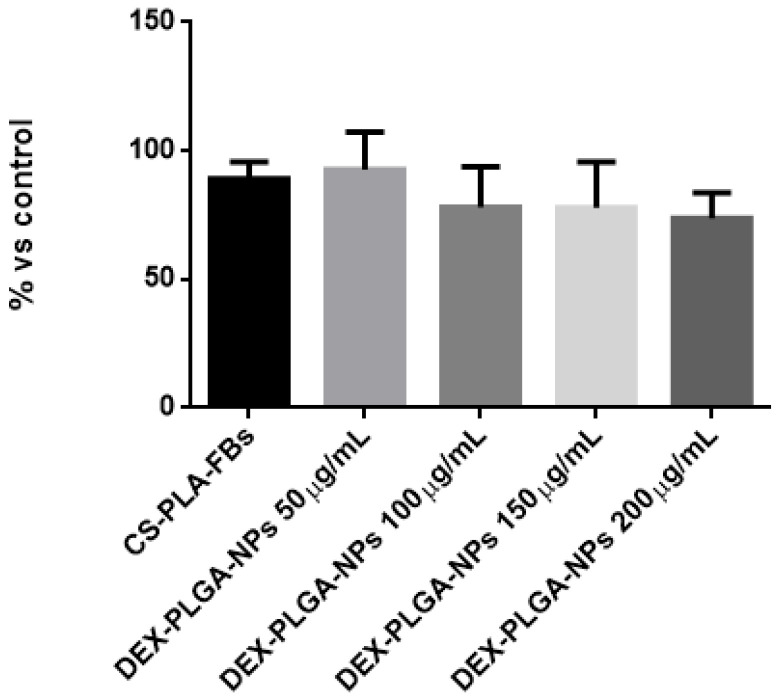
Cell viability of HFs as a result of the MTT assay conducted with CS-PLA-FBs and with PLGA-NPs with different concentrations of DEX.

Regarding putative concerns about PLA-FBs’ cytotoxicity on HFs, we performed a cytotoxicity test by treating HFs with empty CS-PLA-FBs and with different concentrations of DEX-PLGA-NPs (Figure 10).

At the tested concentrations, the viability was higher than 70%, indicating a mild cytotoxic effect, and there were no statistically significant differences between the various treatments. Similar results were obtained using 3T3-Swiss fibroblasts.

### 3.6. Intracellular Concentrations of DEX from PLGA-NPs

In order to verify the drug delivery efficacy of the system, calibration curves were performed before each chromatographic assay to determine the drug concentrations.

After 4 h of incubation with the DEX-PLGA-NPs, a percentage of drug equal to 2.1% ± 0.41 was found inside the cells (Table 4). As regards the incubation with DEX-f, after 4 h its intracellular concentration was equal to 2.25% ± 0.78 (Table 4).

**Figure 11:**
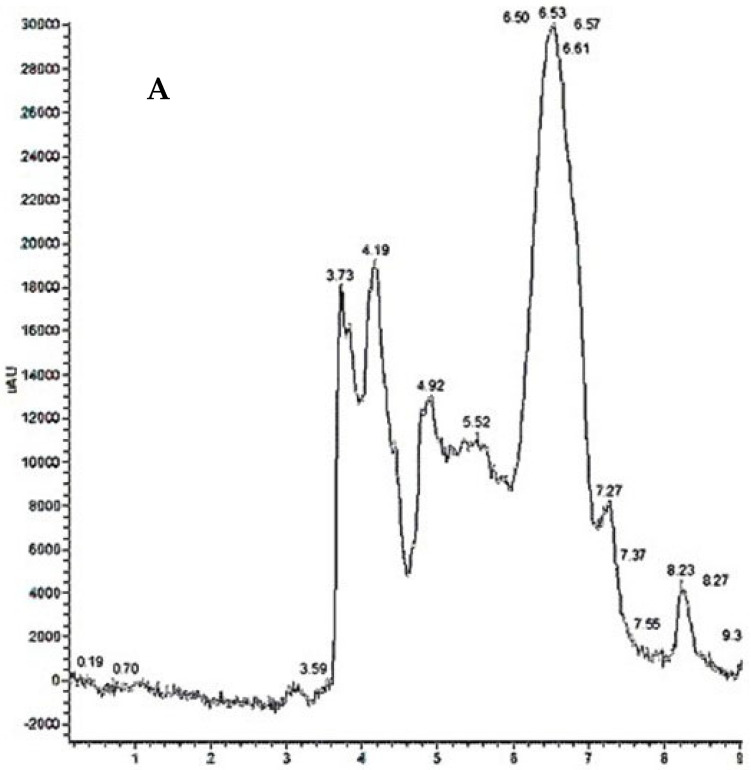

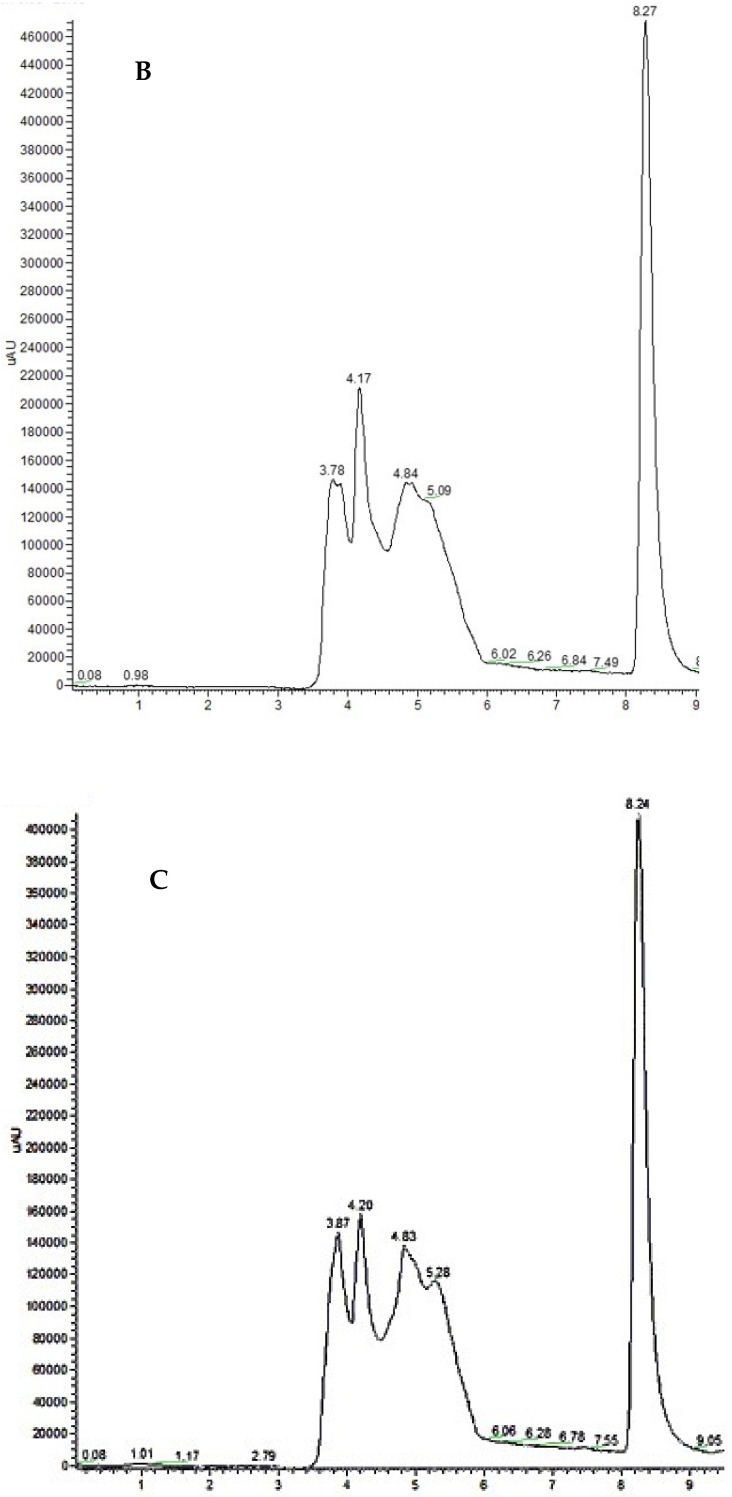
(**A**) Cellular lysate derived from untreated cells. (**B**) Cellular lysate derived from cells treated with DEX-f eluted at RT 8.27 min. (**C**) Cellular lysate derived from cells treated with DEX-loaded PLGA-NPs eluted at RT 8.24 min.

The chromatograms relative to the experiments are reported in Figure 11.

### 3.7. Mucin–CS-PLA-FB Interaction

To evaluate the mucoadhesive properties of the developed delivery systems, mucin was utilized in this study. As a glycoprotein, mucin is a primary component of mucus, contributing to the protective barrier on mucosal surfaces. The interaction between mucin and mucoadhesive materials, such as chitosan, provides crucial insights into the potential of these systems to adhere to mucosal tissues, enhancing drug retention and therapeutic efficacy.

Chitosan, a cationic polymer, interacts with mucin primarily through electrostatic interactions. The positively charged amino groups of chitosan form ionic bonds with the negatively charged sialic acid and sulfate groups on mucin. Additionally, hydrogen bonding and hydrophobic interactions may further contribute to the binding affinity between chitosan and mucin. These interactions strengthen the mucoadhesive properties of chitosan-coated systems, enabling prolonged contact with mucosal surfaces and improving drug delivery performance [44].

**Figure 12:**
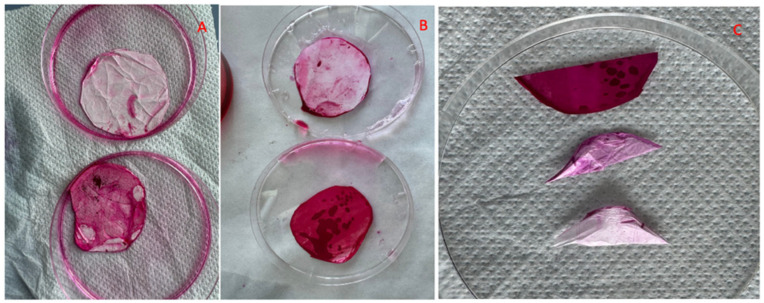

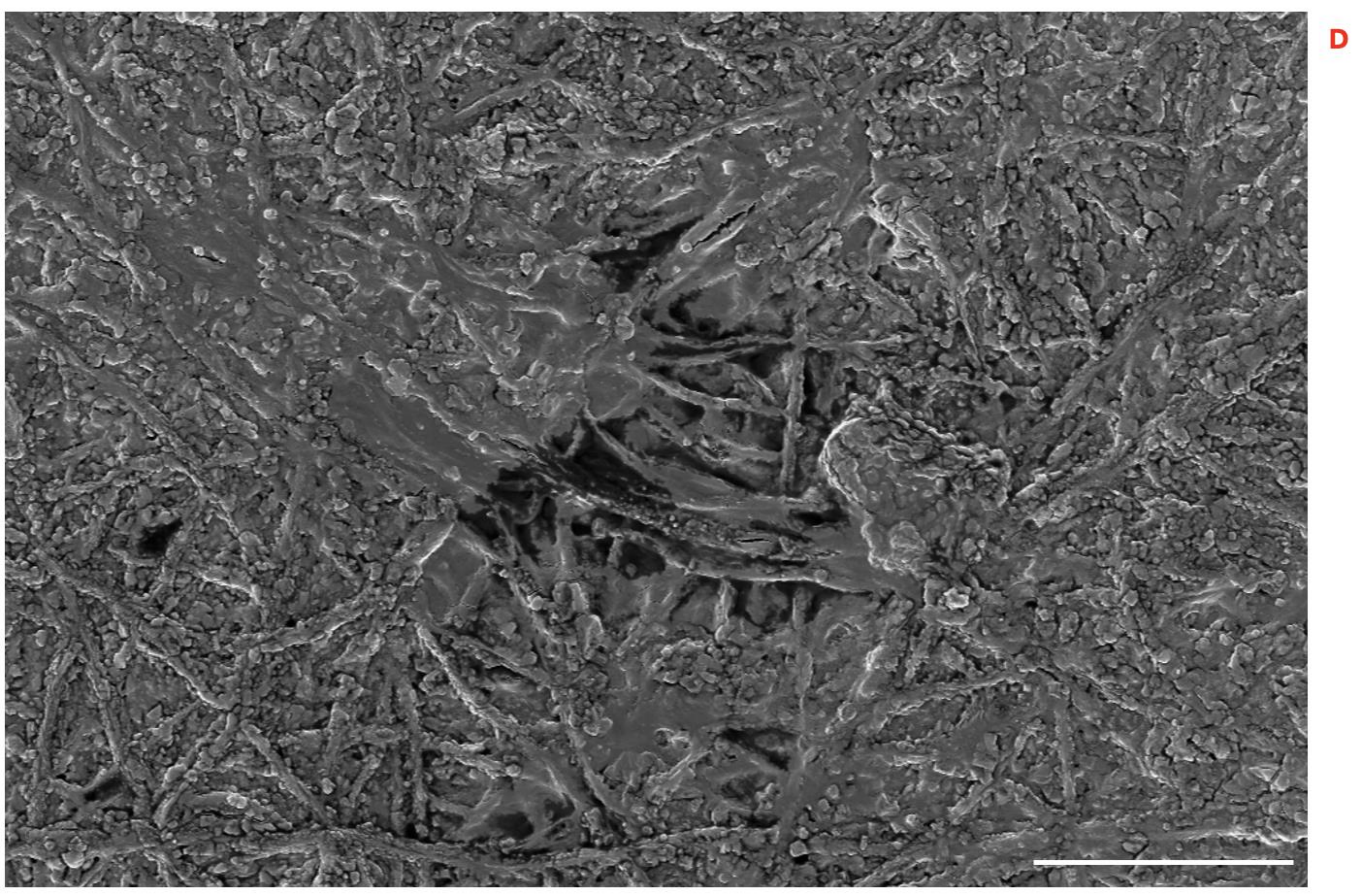
(**A**) PLA-FBs (top) and CS-PLA-FBs (bottom) incubated with 0.4 mg/mL of mucin. (**B**) PLA-FBs (top) and CS-PLA-FBs (bottom) incubated with 2 mg/mL of mucin. (**C**) Comparison of the different colorations obtained after PAS staining between CS-PLA-FBs incubated with mucin (top), PLA-FBs incubated with mucin (middle), and CS-PLA-FBs not incubated with mucin (bottom). (**D**) SEM micrograph of CS-PLA-FBs after incubation with mucin. Scale bar 20 µm.

The PAS assay results revealed a strong coloration in the CS-PLA-FBs incubated with mucin, with the intensity of the coloration increasing alongside higher mucin concentrations (Figure 12A,B). In contrast, the PLA-FBs incubated with mucin showed only a faint coloration (Figure 12A,B), similar to the CS-PLA-FBs that were not exposed to mucin (Figure 12C).

Furthermore, SEM micrographs clearly demonstrated the interaction between the CS-PLA-FBs and mucin. In particular, Figure 12D shows that the initially smooth surface of the fibers becomes coated with an irregular layer after exposure to mucin, indicating mucin adhesion to the fibers.

### 3.8. PLGA-NP Integration into PLA-FBs and Release in DMEM

**Figure 13:**
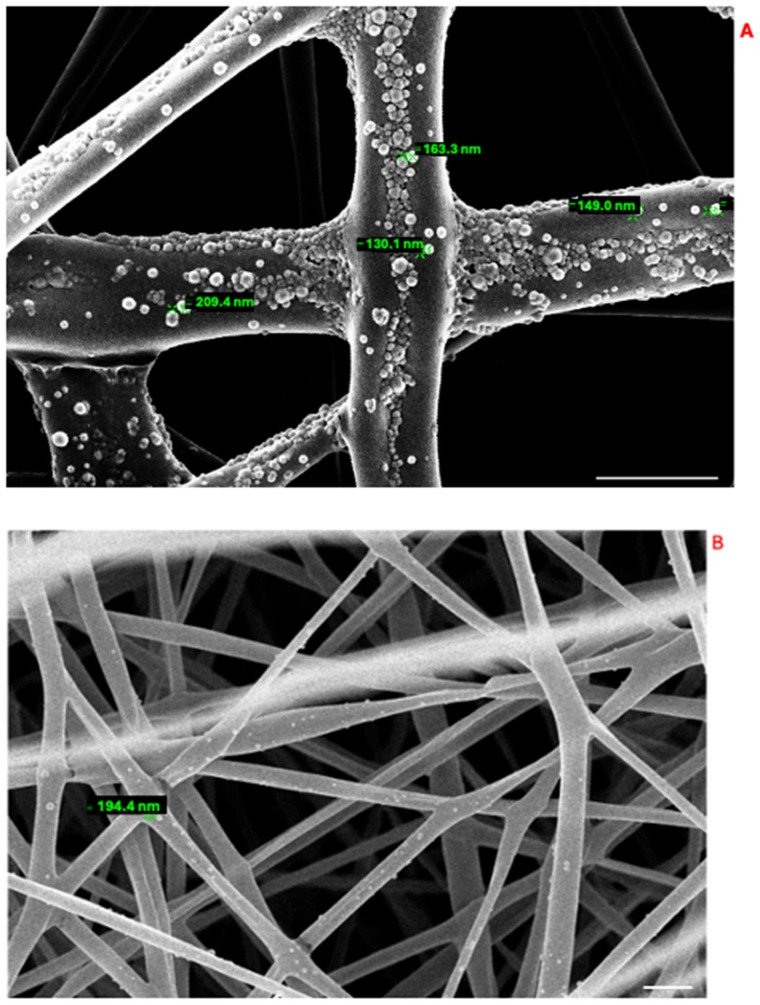
(**A**) SEM images of NPs inside PLA-FBs. Magnification: 31,610×. (**B**) Magnification: 13,630×. Scale bar: 2 µm.

To enhance the functionality of the delivery system, we decided to integrate PLGA-NPs into the PLA-FBs. This approach aimed to combine the mucoadhesive potential arising from the interaction between CS and mucin with the controlled drug release capacity of the NPs. SEM analysis results, reported in Figure 13, showed a uniform distribution of NPs on the PLA-FB surfaces. The evaluation of the NP dimension was coherent with the NP diameter measured by DLS.

**Figure 14:**
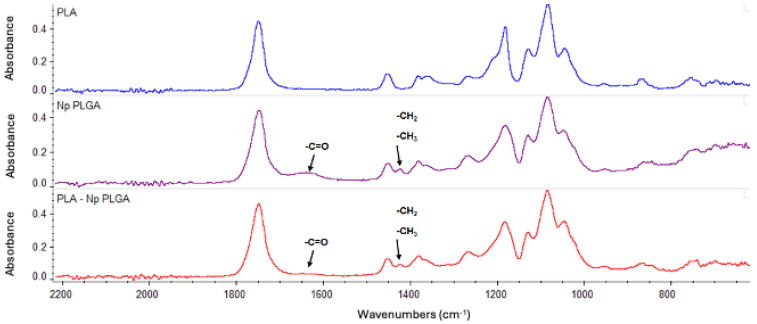
FTIR spectra of PLA fibers, before and after the PLGA NP entrapment, and PLGA NPs.

The effective deposition of PLGA NPs on the surface of PLA-FBs was further confirmed by FTIR measurements. Figure 14 compares the infrared spectra of PLGA nanoparticles and electrospun PLA-FBs before and after PLGA nanoparticle entrapment. In the case of the combined system (PLA fibers decorated with PLGA nanoparticles), it was possible to detect the peak at around 1430 cm^−1^, ascribed to C-H stretching, as well as a broad band at around 1650 cm^−1^, associated with carbonyl -C=O stretching, both typical of PLGA NPs, in addition to the vibrational modes of PLA: the main peaks at around 1750 cm^−1^ and 1088 cm^−1^ correspond to the stretching vibration of the carbonyl group (C=O) and to the stretching vibration of the C-O-C group, respectively [45].

**Figure 15:**
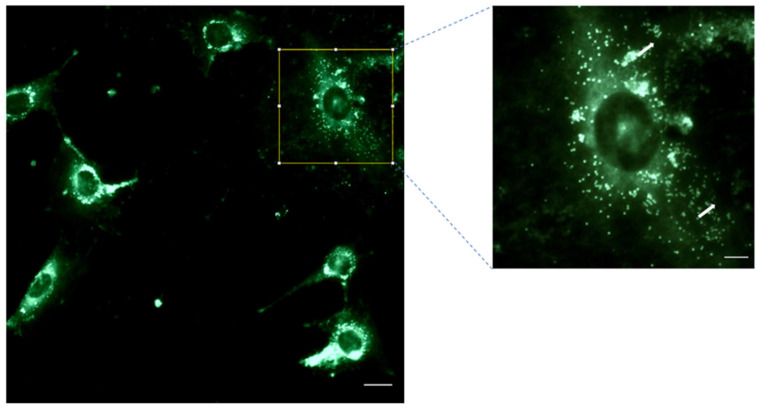
PLGA NPs inside 3T3-Swiss fibroblasts. Scale bar: 20 μm. Zoom: NP details. Scale bar: 260 μm.

In Figure 15, the image of the cells after 1 h of incubation with PLGA-NPs loaded with the fluorescence probe coumarin is shown. The presence of fluorescent NPs inside the cells is evident, highlighting that the NPs had detached from the fibers in which they were originally included and had entered the cells.

### 3.9. Proliferation of 3T3 Swiss Fibroblasts and HFs on PLA Scaffolds and DEX Intracellular Concentrations

**Figure 16:**
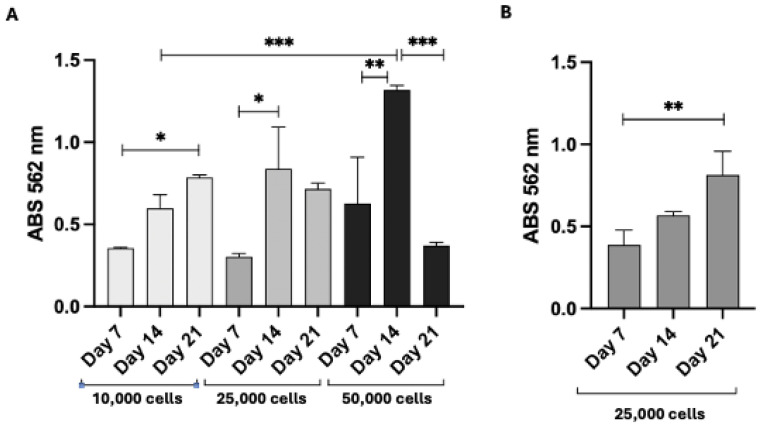
(**A**) Proliferation of 3T3-Swiss fibroblasts on PLA scaffolds, with cells seeded at different densities. * *p* < 0.05; ** *p* < 0.01; *** *p* < 0.001. (**B**) HF proliferation on PLA scaffolds. ** *p* < 0.01.

Next, we evaluated the cell proliferation after seeding cells on PLA scaffolds to determine the optimal conditions for cell growth. As illustrated in Figure 16A, 3T3 Swiss fibroblasts grow uniformly across all conditions up to 14 days. Beyond this period, cell proliferation becomes dependent on the initial seeding density. For experiments extending to 21 days, 10,000 cells per scaffold are recommended, while 25,000 cells per scaffold are sufficient for 7- or 14-day experiments. Consequently, the initial cell seeding density should be chosen based on the experimental duration. For this reason, the experiments with HFs were carried out with 25,000 cells per scaffold for a 14-day duration.

As depicted in Figure 13B, the rise in Abs values confirms that HFs successfully proliferate on PLA scaffolds. Significant differences in Abs were observed between day 7 and day 21, reflecting sustained cell growth over time. These results validated the use of the same scaffolds in subsequent studies under dynamic conditions to explore drug release and cellular uptake.

DEX released from the NPs revealed that the amount of drug inside the cells was 2.1% of the total amount loaded into the particles. This result is the same as that obtained under static conditions.

## 4. Discussion

The oral cavity is a strategic site for drug administration, offering various advantages like bypassing the gastrointestinal tract and hepatic portal system, enhancing patient compliance, sustaining drug delivery, enabling rapid pharmacological action, and simplifying drug application and removal [46,47]. However, the effectiveness of drug delivery in this region is significantly limited by the characteristics of the oral mucosa, which is protected by viscoelastic mucus layers. These layers hinder the adhesion of topical drugs, leading to their rapid clearance and limiting their ability to penetrate and reach the epithelial surface [48]. Additional factors like salivary flow, swallowing, chewing, and phonation further reduce drug retention time and therapeutic efficacy [49]. To counter these limitations, numerous drug delivery systems (DDSs) are being investigated to ensure prolonged and effective drug delivery to the mucosa [20,50].

The interaction time between a drug formulation and the oral mucosa can be enhanced using mucoadhesive materials, which is a focus of many research efforts aiming to improve the efficacy of various DDSs [23,24]. However, despite significant research on oral mucosal drug delivery, only a few commercially available formulations are specifically designed for this route [18]. This gap highlights the need for further innovation in the development of effective DDSs.

Current buccal DDSs include tablets, films, patches, semisolids/liquids, and particulates [51]. Mucoadhesive buccal tablets are designed to adhere to the mucosa, maintaining their position despite saliva and movement, although their rigidity may lead to lower patient compliance for prolonged use [52,53]. Patches, on the other hand, consist of multiple layers, including a waterproof backing, a drug reservoir, and a bioadhesive surface, while films are flexible and soft but require significant bioadhesive power. Although gels and ointments are easy to apply, they offer less precise drug dosing compared to other forms like tablets, patches, or films [20,54]. Each delivery form has its own set of advantages and challenges that influence both efficacy and patient acceptance. However, recent advancements in nanotechnology are enabling the creation of carriers that can effectively address these challenges while minimizing the side effects associated with drugs.

In this study, a dual DDS was developed and characterized. It is composed of electrospun fibers made of PLA [55], coated with chitosan and enriched with PLGA-NPs loaded with DEX. Both the CS-PLA-FBs and PLGA-NPs were characterized using SEM. The acquired micrographs revealed that CS formed a homogeneous layer on the fibers, while the uncoated fibers exhibited a smooth surface with no imperfections. Additionally, the average fiber diameter was assessed.

The size of the PLGA-NPs was characterized using DLS and SEM. The results showed homogeneity in both shape (spherical) and size (below 200 nm), with a polydispersity index (PI) of 0.09, indicating the absence of aggregation and meeting the criteria for nanoscale dimensions [56]. The encapsulation efficiency was 50%, consistent with the literature, likely due to the lipophilic nature of the compound.

The drug release capability of nanoparticles was evaluated in ethanol, demonstrating that NPs release the drug with an initial burst of about 90% within the first hour, confirming the lack of strong bonds between the carriers and the drug.

Before assessing the drug release efficiency in cells, the cytocompatibility of the PLGA-NPs was first investigated. This was carried out using 3T3-Swiss fibroblasts and fibroblasts isolated from OLP patients (HFs) to ensure consistency in the results. Cytocompatibility assays were conducted with both empty and DEX-loaded PLGA-NPs, selecting a drug concentration of around 70 µg/mL. This concentration was selected because it mirrors therapeutic levels without showing toxicity effects. As expected, given the well-known biocompatibility of all the compounds used, no toxic effect was observed or, at most, a slight cytotoxicity of less than 30%.

At this point, the uptake of NPs and the release of DEX within cells were investigated. Specifically, to quantify the amount of drug released into the cytoplasm, an HPLC method was developed with a mobile phase consisting of water and CH_3_CN under isocratic conditions. The method was defined in terms of LOD (limit of detection) and LOQ (limit of quantification), which were, respectively, 0.3 µg/mL and 0.92 µg/mL. The recovery efficiency from the cell lysate was also evaluated, showing almost 100%.

Moreover, comparing the intracellular concentration of DEX, both when administered inside PLGA-NPs or as free DEX, it was possible to evidence that NPs are able to release an amount of drug inside the cells comparable to the amount of non-encapsulated DEX that enters by diffusion. However, it is important to note that, at the 4 h time point, no significant difference was observed in drug uptake between the NP-encapsulated DEX and the free DEX. This suggests that, under the experimental conditions, the NPs did not enhance the drug’s penetration. The potential advantage of NPs in this drug delivery system may not lie in increasing the drug’s penetration but rather in offering controlled release, improving drug stability, or enabling targeted delivery, which could be more apparent over longer incubation times or in other experimental conditions.

Both the SEM and the FTIR results relative to the combination of CS-PLA-FBs and PLGA-NPs supported a uniform distribution of the nanoparticles on the FB surfaces. Moreover, the fluorescence microscopy analysis of the CS-PLA-FBs combined with 6-coumarin-loaded PLGA-NPs demonstrated that the PLGA-NPs are able to detach from PLA-FBs and penetrate inside the cells. The mucoadhesive capacity of the system was confirmed by PAS staining and SEM. Fluorescence image analysis confirms that PLGA-NPs successfully enter OLP cells.

This delivery system was also characterized under dynamic conditions with the use of a bioreactor in order to simulate both salivary flow and the three-dimensionality of oral cavity tissue, as fibroblasts taken from a patient were grown on PLA scaffolds, printed by FDM. It is particularly interesting to observe that the particles’ ability to penetrate the cells is not altered by the flow. This result further confirms the validity of a DDS that synergically ensures better mucoadhesion through CS-coated fibers and increases drug penetration via NPs, even in the presence of a flow similar to saliva. Also, toxicity analysis under dynamic conditions did not reveal different results compared to static conditions.

## 5. Conclusions

This study clearly demonstrates that the combination of PLGA-NPs with PLA-FBs coated with chitosan merges the benefits of mucoadhesion and effective drug penetration. The results highlight the potential of such systems for effective clinical application in the delivery of DEX, contributing to more targeted therapy for oral diseases. In fact, the developed DDS offers several innovative features and advantages. In particular, the coating of PLA-FBs with CS significantly enhances the system’s ability to adhere to the oral mucosa, ensuring prolonged contact between the drug and the target site. This results in a more effective localized release of DEX, improving the therapeutic efficacy and reducing the need for frequent applications, thus increasing patient compliance. Another important advantage is that this DDS represents a versatile platform. The system, combining PLGA-NPs embedded in PLA-FBs with a CS coating, can be adapted for the delivery of various drugs. This adaptability allows for the creation of personalized treatments for different oral health conditions, offering a more targeted and customized approach to drug delivery. Furthermore, the soft and flexible nature of PLA-FBs is expected to provide a comfortable experience for patients. The softness of the fibers minimizes the risk of irritation or discomfort in the sensitive tissues of the oral cavity, making the system more acceptable and enhancing patient adherence to treatment regimens. These features underscore the innovation and patient-centric design of the developed delivery system, making it a promising solution for improving oral cavity treatments. To further enhance clinical application, it is crucial to optimize the system to achieve a balance between drug release and mucoadhesive properties. Future research will focus on improving this delivery system and providing a detailed analysis of its physicochemical characteristics, including drug release in cells. These actions are important to achieve optimal treatment effectiveness and improve patient compliance, ultimately contributing to the development of new effective approaches for treating oral diseases.

## Figures and Tables

**Table 1:** Statistical analysis of the cytotoxicity measures induced by the different concentrations of DEX-f.

Tukey’s Multiple Comparisons Test	*p* Value
DMSO vs. 100.0 µg/mL	>0.05
DMSO vs. 50.0 µg/mL	>0.05
DMSO vs. 25.0 µg/mL	>0.05
DMSO vs. 12.5 µg/mL	>0.05
DMSO vs. 6.0 µg/mL	>0.05
DMSO vs. 3.0 µg/mL	>0.05

**Table 2:** Linear regression equation and LOD and LOQ values.

	DEX
Linear regression equation	Y = 3.29 × 10^6^ X + 11,437
DEX concentration range used for the calibration curve	From 0.625 to 5 µg/mL
R^2^	0.99
LOD	0.3 µg/mL
LOQ	0.92 µg/mL

**Table 3:** DEX recovery into 3T3 Swiss Fibroblasts.

	Drug Added to Cell Lysates (µg/mL)	Drug Recovery from Cell Lysates (µg/mL)	%
DEX	1.85	1.79	96.7
DEX	1.72	1.68	97.7

**Table 4:** DEX found inside HFs after four hours of incubation with DEX-loaded PLGA NPs and with DEX-f.

Intracellular DEX	µg DEX	% of DEX vs. Total DEX
NPs 4 h	14.70 ± 2.87	2.1 ± 0.41
DEX-f 4 h	15.75 ± 5.46	2.25 ± 0.78

## Data Availability

The original contributions presented in this study are included in the article. Further inquiries can be directed to the corresponding author.
